# Synergistic Anticancer Effects of Vorinostat and Epigallocatechin-3-Gallate against HuCC-T1 Human Cholangiocarcinoma Cells

**DOI:** 10.1155/2013/185158

**Published:** 2013-06-23

**Authors:** Tae Won Kwak, Do Hyung Kim, Chung-Wook Chung, Hye Myeong Lee, Cy Hyun Kim, Young-IL Jeong, Dae Hwan Kang

**Affiliations:** National Research and Development Center for Hepatobiliary Disease, Pusan National University Yangsan Hospital, Gyeongnam 626-770, Republic of Korea

## Abstract

The aim of this study was to investigate the effect of the combination of vorinostat and epigallocatechin-3-gallate against HuCC-T1 human cholangiocarcinoma cells. A novel chemotherapy strategy is required as cholangiocarcinomas rarely respond to conventional chemotherapeutic agents. Both vorinostat and EGCG induce apoptosis and suppress invasion, migration, and angiogenesis of tumor cells. The combination of vorinostat and EGCG showed synergistic growth inhibitory effects and induced apoptosis in tumor cells. The Bax/Bcl-2 expression ratio and caspase-3 and -7 activity increased, but poly (ADP-ribose) polymerase expression decreased when compared to treatment with each agent alone. Furthermore, invasion, matrix metalloproteinase (MMP) expression, and migration of tumor cells decreased following treatment with the vorinostat and EGCG combination compared to those of vorinostat or EGCG alone. Tube length and junction number of human umbilical vein endothelial cells (HUVECs) decreased as well as vascular endothelial growth factor expression following vorinostat and EGCG combined treatment. These results indicate that the combination of vorinostat and EGCG had a synergistic effect on inhibiting tumor cell angiogenesis potential. We suggest that the combination of vorinostat and EGCG is a novel option for cholangiocarcinoma chemotherapy.

## 1. Introduction

Cholangiocarcinoma, which arises from biliary tract epithelia, is a rare malignancy associated with poor prognosis and high mortality [1–3]. The incidence rate of cholangiocarcinoma has increased worldwide [4–6]. However, the reason for the increase in cholangiocarcinoma remains unclear. Cholangiocarcinoma accounts for 3% of all gastrointestinal cancers and for approximately 15% of liver cancers worldwide [4–7]. Cholangiocarcinoma progresses insidiously and is difficult to diagnose. Although surgical resection is a unique option for curative treatment of cholangiocarcinoma, patients diagnosed with cholangiocarcinoma are frequently identified in an advanced stage and cannot be practically considered for surgical resection. Palliative therapies such as chemotherapy, radiotherapy, displacement of drug-eluting stents, and photodynamic therapy can be considered to increase patient quality of life. Although cholangiocarcinoma scarcely respond to traditional chemotherapy [8, 9] and has a poor prognosis, chemotherapy remains a feasible treatment option for cholangiocarcinoma. Most chemotherapeutic agents such as cisplatin, oxaliplatin, and gemcitabine inhibit cancer cell proliferation. However, systemic treatment of cholangiocarcinoma with these kinds of anticancer agents is always unsuccessful. Thus, a novel treatment option is required, as progression of cholangiocarcinoma is dependent on invasion, metastasis, spreading, and proliferation [1, 10–12].

Acetylation/deacetylation of histones plays an important role in the transcriptional regulation of cells, and histone deacetylase inhibitor (HDACi) has been investigated as a promising new class of cancer chemotherapeutic drugs [13–15]. A number of HDACis are in clinical trials for various neoplastic diseases. HDACis modify gene transcription, by chromatin remodeling and by changes in the structure of proteins in transcription factor complexes [15, 16]. Vorinostat (suberoylanilide hydroxamic acid) was the first HDACi approved by the US Food and Drug Administration for clinical use to treat advanced cutaneous T cell lymphoma. Vorinostat induces differentiation, growth arrest, and apoptosis of various tumor cells in culture [14, 15]. Furthermore, vorinostat has antiangiogenic activity [17].

The beneficial effect of (−)-epigallocatechin-3-gallate (EGCG), which is a major polyphenolic constituent of green tea, has been reported for various kinds of cancer [18–23]. EGCG is a potent antioxidant and has an anti-proliferative effect against tumors without adverse harmful effects on normal cells [21, 24]. EGCG regulates expression of VEGF, MMPs, PA, insulin-like growth factor, epidermal growth factor receptor, and cell cycle regulatory proteins and inhibits NF-*κ*B, PI3-K/Akt, Ras/Raf/mitogen activated protein kinase, and activator protein 1 signaling pathways [18]. These biological activities produce meaningful chemopreventive effects against cancer. In particular, EGCG has anti-invasive and antimetastatic effects on cancer cells [25–27]. EGCG also modulates acetylation of the androgen receptor by antihistone acetyltransferase activity and then suppresses prostate cancer cell growth [28].

 In this study, we investigated the combined synergistic effect of vorinostat and EGCG against HuCC-T1 human cholangiocarcinoma cells *in vitro*. A synergistic chemopreventive effect against human cholangiocarcinoma cells was expected because vorinostat and EGCG have anti-proliferative, anti-angiogenic, and anti-invasive effects [14, 17, 19–28]. We studied the combined effect of vorinostat and EGCG for the apoptosis, cytotoxicity, invasion, and angiogenic potential of HuCC-T1 cells *in vitro*.

## 2. Material and Methods

### 2.1. Preparation of Materials and Reagents

EGCG was purchased from Sigma Chem. Co. (St. Louis, MO, USA) and was dissolved in DMSO (20 mg/mL) as a stock solution. Vorinostat was purchased from LC Lab Co. and was dissolved in DMSO (50 mg/mL). RPMI1640 media, fetal bovine serum (FBS), and all cell culture components were purchased from Life Technologies (Grand Island, NY, USA). All reagents used were extrapure grade.

### 2.2. Cell Cultures

HuCC-T1 cholangiocarcinoma cells were obtained from the Health Science Research Resources Bank (Osaka, Japan) and maintained with RPMI1640 medium supplemented with 10% heat-inactivated FBS and 1% penicillin/streptomycin at 37°C in a humidified atmosphere containing 5% CO_2_.

### 2.3. Trypan Blue Exclusion Assay (Inhibition of Cell Growth and Cytotoxicity Assay)

HuCC-T1 cells were seeded in 24-well plates at a density of 3 × 10^4^ and 3 × 10^5^ cells per well for growth inhibition and cytotoxicity assay, respectively. And then, each well plate was incubated overnight in a CO_2_ incubator. Vorinostat and EGCG in DMSO were diluted with RPMI1640 media containing 10% FBS for growth inhibition assay at various concentrations and then added to tumor cells in 24-well plates following a 24-, 48-, and 72-hours incubation. And cytotoxicity assay was measured using serum-free RPMI1640 media. The control was treated with 0.1% (v/v) DMSO. The cells were trypsinized, harvested, and resuspended in PBS. Trypan blue was added, and then the number of cells was counted using the Countess Automated Cell Counter (Invitrogen, Carlsbad, CA, USA). Inhibition of tumor cells growth was determined as the percentage of treated cells versus control cells.

### 2.4. Annexin V/Propidium Iodide (PI) Binding Assay

HuCC-T1 cells were seeded in 6-well plates at a density of 1 × 10^6^ cells per well and exposed to various concentrations of vorinostat and EGCG for 24 hours. The cells were harvested, washed with PBS, resuspended in 100 **µ**l binding buffer, and stained with FITC-conjugated Annexin V for the apoptosis analysis and with PI for the necrosis analysis. These cells were analyzed by flow cytometry.

### 2.5. Protein Lysates and Western Blot Analysis

Cells were trypsinized and washed with cold PBS. The cells were collected by centrifugation and lysed in lysis buffer containing protease inhibitors (50 mM Tris, 150 mM NaCl, 1% NP-40, 0.5% deoxycholic acid, 0.1% sodium dodecyl sulfate, [SDS]) with phenylmethylsulfonyl fluoride and a protease inhibitor cocktail (Roche Diagnostics, Indianapolis, IN, USA). The cell suspension was cleared by centrifugation at 14,000 ×g for 30 min at 4°C, and then supernatant or cell lysates were collected. Protein concentration was determined using the BCA Protein Assay kit (Pierce, Rockford, IL, USA).

 For Western blotting, 50 **µ**g protein was subjected to SDS-polyacrylamide gel electrophoresis (SDS-PAGE), transferred to a PVDF membrane, blocked with 5% skim milk in TBS-T, then probed with an appropriate primary antibody followed by a secondary HRP-conjugated antibody. Proteins were detected by chemiluminescence. Blots were subsequently stripped and reprobed with anti-*β*-actin primary antibody followed by the appropriate secondary antibody and chemiluminescence detection as a loading control. Proteins were quantified by digital analyses of the protein bands using the ImageJ software program.

### 2.6. Gelatin Zymography

HuCC-T1 cells (1 × 10^6^ cells/well) were seeded in 6-well plates and exposed to various concentrations of vorinostat and EGCG for 24 hours. MMP activity in the conditioned medium was analyzed by substrate-gel electrophoresis using SDS-PAGE containing 10% gelatin (gelatin zymography). Equal volumes of conditioned cell culture medium samples were mixed with Laemmli buffer under nonreducing conditions, loaded onto the gel and separated by electrophoresis. SDS was removed by soaking the gels three times for 30 min at room temperature in Triton buffer (2.5% Triton X-100 in PBS) and the gels were incubated for 24 hours at 37°C. The gels were stained with 0.1% Coomassie Brilliant Blue R-250 and destained until clear bands became evident. Quantitative results of the assays were obtained by densitometry.

### 2.7. TUNEL Assay

Fragmented DNA indicating apoptosis of tumor cells was observed by TUNEL. Cells exposed to various combinations of vorinostat and EGCG for 24 hours were fixed in 4% paraformaldehyde in PBS and then stained with an *in situ* apoptosis detection kit (Millipore, Billerica, MA, USA) according to manufacturer's protocol. Briefly, slides were incubated with 3% H_2_O_2_ for 10 minutes at room temperature to block endogenous peroxidase activity and then with the TdT enzyme in a humidified chamber at 37°C for 1 hour. Subsequently, the slides were incubated with blocking reagent in a humidified chamber at 37°C for 30 minutes and then incubated with anti-DIGOiygenin-biotin (1 : 100) at 37°C for 30 minutes. After the streptavidin-biotin complex (SABC, 1 : 100) was applied to the slides at 37°C for 30 minutes, immunoreactivity was visualized with the Alexa488 secondary antibody. The negative control used distilled water in place of the TdT solution. The presence of clear nuclear staining was indicative of apoptotic cells. At least 500 cell nuclei were examined in the most evenly and distinctly labeled areas. The number of TUNEL-positive tumor cell nuclei was counted, and the apoptotic index was the percentage of apoptotic cells in the tumor.

### 2.8. Cell Invasion Assay

The invasion assay was performed as reported previously [12]. Transwell chambers in 24-well plates were employed to measure invasive potential of tumor cells. The upper chamber was coated with 20 *μ*L Matrigel (1 mg/mL; BD Bioscience, San Jose, CA, USA). HuCC-T1 cells were seeded on the upper parts of the Transwell chamber at 2 × 10^4^ cells in 100 *μ*L serum-free medium with or without vorinostat and EGCG. The chamber was placed into the 24-well plate, which contained 600 *μ*L of RPMI1640 containing 10% FBS. These cultures were incubated for 24 hours at 37°C in a CO_2_ incubator. Cells on the upper surface of the membrane were fixed with methanol, stained with hematoxylin and eosin, and photographed at the end of the incubation. Cells from various areas of the lower surface of the membrane were counted using a computerized video image analyzing system. Each assay was performed in triplicate, and mean ± standard deviation is presented.

### 2.9. Wound Healing Assay

A wound healing assay of HuCC-T1 CC cells was performed using a wound healing assay kit containing ibidi Culture-Inserts (ibidi GmbH, Planegg/Martinsried Germany) as reported previously [12]. Aliquots containing 5 × 10^5^ cells in RPMI1640 supplemented with 10% FBS were seeded on 6-well plates and the cells were exposed to vorinostat and EGCG at 37°C and 5% CO_2_ for 24 hours. The cells were then washed twice with PBS and harvested by trypsinization. Then, 5 × 10^4^ cells in serum free RPMI1640 were seeded into the culture inserts following a 24-hour incubation. The wound healing and migrated cell zone was observed using light microscopy. Serum-free medium was used to avoid proliferation-dependent migration of tumor cells.

### 2.10. Antiangiogenesis Assay

An angiogenesis assay was performed as reported by Okabe et al. [29]. When HuCC-T1 cells filled dishes to 70–80% confluency, the medium was replaced with serum-free RPMI1640 and then cells were treated with vorinostat and EGCG for 24 hours. The media were centrifuged at 1000 rpm for 5 min and the supernatant was used as the medium. Protein content of the medium was determined with a BCA Assay kit (Pierce) and aliquots were stored at −80°C until use. HUVECs (1 × 10^4^ cells/well) were suspended in a mixture of Medium/EGM-2 medium (1 : 1) with 0.5% FBS and then seeded on 50 *μ*L of Matrigel in 96-well plates. These plates were incubated for 12 hours, and cell morphology was examined in each well. The total capillary tube length and branching points were examined in three random view fields per well, and the average values were calculated.

### 2.11. Statistical Analysis

Statistical analyses of the data between treated and untreated cells were performed using Student's *t*-test. A *P* value < 0.05 was considered significant. ∗ represents statistical significance compared to the vehicle-treated control.

## 3. Results

### 3.1. Inhibition of Cell Growth and Cytotoxic Effect of Vorinostat and EGCG on the HuCC-T1 Cell Line

**Figure 1:**
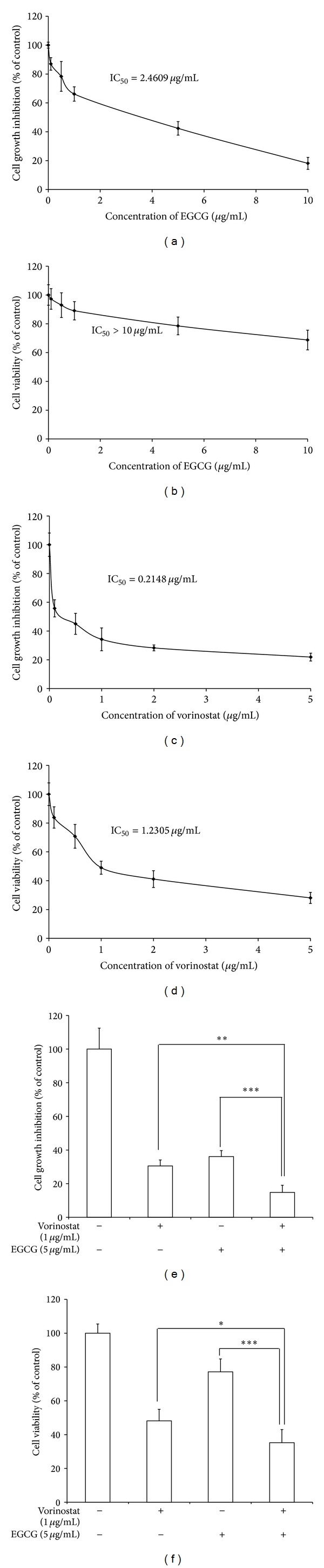
The synergistic anticancer effects of vorinostat and EGCG were measured by cytotoxic and growth inhibition responses. A 3 × 10^4^ aliquot of cells for the cell cytotoxicity assay and 3 × 10^3^ cells for growth inhibition assay were seeded in 96-well plates. RPMI1640 media supplemented 10% FBS was used to asses tumor cell growth inhibition, whereas serum-free media were used for cell cytotoxicity assay. Single agent treatment: (a) and (b), EGCG; (c) and (d), vorinostat. Combined vorinostat and EGCG: (e) and (f). Growth inhibition: (a), (c), and (e). Cell cytotoxicity: (b), (d), and (f). *P < 0.05; **P < 0.01; ***P < 0.001.

Tumor cell proliferation and viability were determined using the Trypan blue dye exclusion assay to identify the synergistic effect of the combination of vorinostat and EGCG. The IC_50_ values of the vorinostat and EGCG treatment alone were 2.4609 and 0.2148 *μ*g/mL, respectively. One and 5 *μ*g/mL vorinostat were used to test the combined effect with EGCG. As shown in Figure 1(e), synergistic inhibition of growth (85%) was observed with the vorinostat and EGCG combination, whereas each agent alone resulted in 70% inhibition for EGCG and 75% for vorinostat. A synergistic cellular cytotoxicity effect was also observed with the combined vorinostat and EGCG treatment, as shown in Figure 1(f). EGCG did not have a cytotoxic effect because ≥60% and 78% cells survived at 10 and 5 *μ*g/mL EGCG, respectively. As shown in Figure 1(d), the vorinostat IC_50_ was 1.2305 *μ*g/mL. Figure 1(f) showed that significant toxicity of up to 52% with vorinostat and 23% with EGCG was observed. The vorinostat and EGCG combination caused significantly greater cholangiocarcinoma toxicity (65%) (Figure 1(f)).

### 3.2. Apoptosis and Necrosis of HuCC-T1 Cholangiocarcinoma Cells following Vorinostat and EGCG Treatment

**Figure 2:**
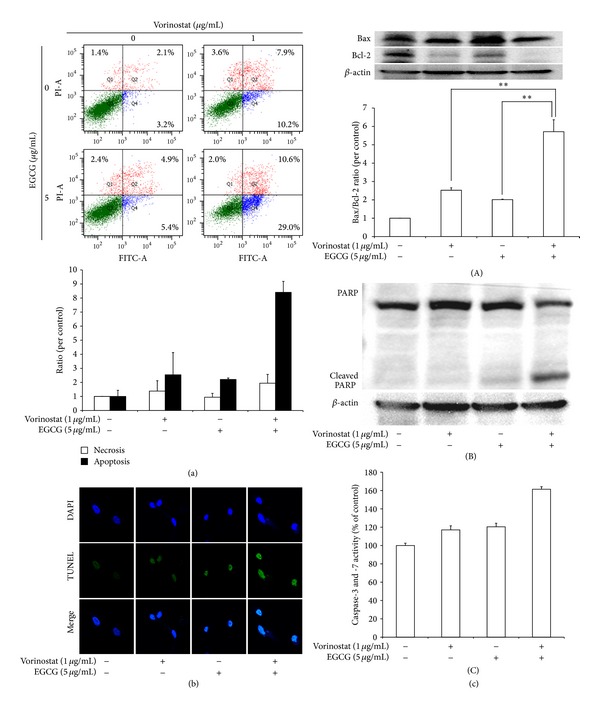
The synergistic effect of the combination of vorinostat and EGCG was assessed on apoptosis and necrosis of HuCC-T1 cholangiocarcinoma cells. (a) Flow cytometric analysis of tumor cells. FITC-Annexin V and propium iodide (PI) were used for apoptosis and necrosis analysis of tumor cells, respectively. (b) TUNEL assay. (c) Western blot assay: (A), Bax/Bcl-2 expression; (B), PARP expression; extent of caspase-3 and-7 activity. **P < 0.01.

Figure 2 shows the apoptosis and necrosis analysis of HuCC-T1 cholangiocarcinoma cells in response to vorinostat and EGCG. As shown in Figure 2(a), both of vorinostat and EGCG induced tumor cell apoptosis rather than necrosis. In particular, the vorinostat and EGCG combination significantly enhanced apoptosis in HuCC-T1 cells. The apoptosis index of the vorinostat and EGCG combination was approximately three fold higher than that when vorinostat or EGCG was treated alone. These results indicate that the combination of vorinostat and EGCG induced synergistic anticancer effects on tumor cells. Furthermore, the TUNEL assay showed that the vorinostat and EGCG combination had a synergistic anticancer effect. Fluorescence intensity in the combined vorinostat and EGCG treatment was obviously higher than that of the single treatments. The Bax/Bcl-2 expression ratio increased significantly twofold compared to individual treatment following treatment with the combination of vorinostat/EGCG. PARP, a protein involved in DNA repair and programmed cell death, also decreased following treatment with the vorinostat and EGCG combination, whereas the single treatments showed obvious increased protein intensities for both vorinostat and EGCG alone. Furthermore, caspase-3 and -7 activity of the combination was also higher than that of the single treatments.

These results indicate that the vorinostat and EGCG combination induced a synergistic anti-cancer effect against HuCC-T1 human cholangiocarcinoma cells.

### 3.3. Anti-Invasion, Antimigration, and Antiangiogenesis Effects

**Figure 3:**
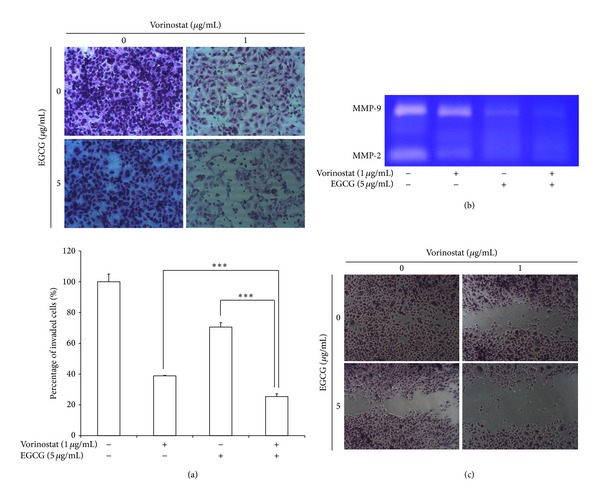
The combined effect of vorinostat and EGCG was assessed on invasion and migration capacity of HuCC-T1 cholangiocarcinoma cells. (a) Matrigel invasion assay. (b) Gelatin zymography: MMP-2 and -9 expression. (c) Wound healing assay for tumor cell migration. ***P <0.001.

**Figure 4:**
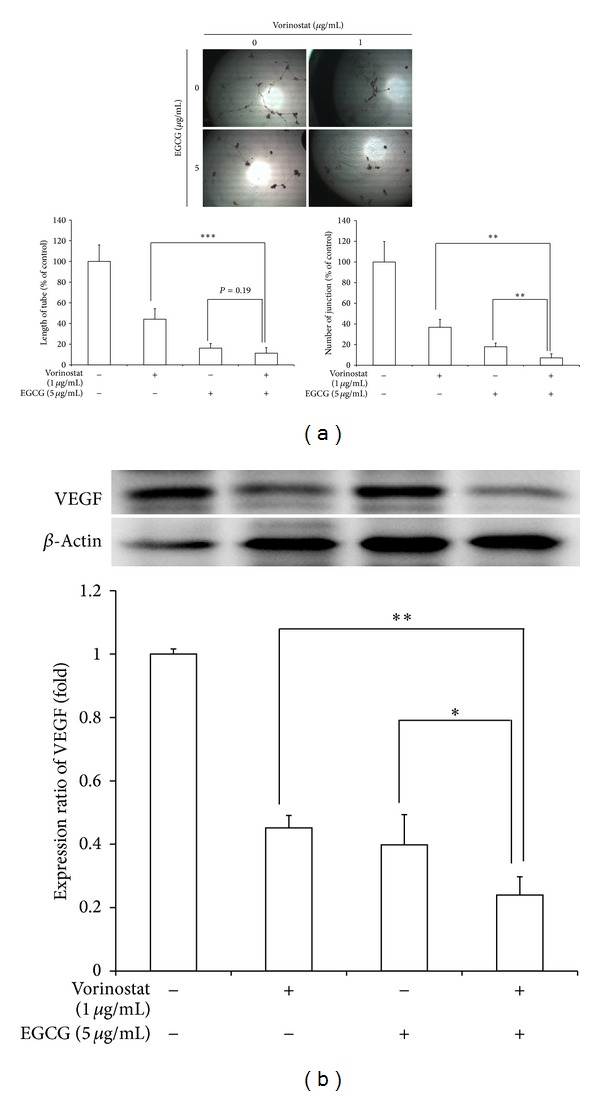
The combined effect of vorinostat and EGCG was assessed on angiogenic activity of tumor cells. HUVECs were used for the angiogenesis assay. Treated media from tumor cell cultured with the combination of vorinostat and EGCG were added to HUVECs. (a) The length of tubes and the numbers of junction were counted and compared with those of the control. (b) Western blot assay: VEGF expression and ratio. *P < 0.05; **P < 0.01; ***P < 0.001.

Figures 3 and 4 show invasion, migration, and angiogenesis behavior of HuCC-T1 human cholangiocarcinoma cells with single and combined treatment. The tumor cell invasive potential was assessed by Matrigel invasion assay. As shown in Figure 3(a), vorinostat/EGCG combinations were markedly inhibited invasion of tumor cells compared to single treatment of vorinostat or EGCG; that is, tumor cell invasive capacity was inhibited 13% versus vorinostat single treatment and 45% versus EGCG single treatment. Furthermore, MMP-2 and -9, which play a crucial role in basement membrane degradation and tumor cell invasion, also decreased following the combined vorinostat and EGCG treatment and the expression level of MMP-2 or -9 was significantly lower at drug combination than single treatment. The wound healing assay was employed to study the effect of the combined vorinostat and EGCG treatment on tumor cell migration potential. Migration potential of HuCC-T1 cholangiocarcinoma cells was also obviously inhibited by the combined vorinostat and EGCG treatment compared to single treatment (Figure 3(c)).

Figure 4 shows the anti-angiogenesis effect of the vorinostat and EGCG combination in HUVECs. As shown in Figure 4(a), treated media from tumor cell cultures treated with the combination of vorinostat and EGCG or the agents alone showed the highest capacity to inhibit HUVEC tube and junction formation. Furthermore, VEGF was suppressed by the combined treatment (Figure 4(b)).

These results indicated that a combination of vorinostat and EGCG has synergistic effects to suppress invasion and migration of HuCC-T1 cells and suppress the angiogenesis potential of HUVECs.

## 4. Discussion

 Conventional chemotherapeutic agents have very low therapeutic efficacy for cholangiocarcinoma [1, 10–12]. Although palliative therapies such as chemotherapy, radiotherapy, drug-eluting stents, and photodynamic therapy have beneficial effects as cholangiocarcinoma treatments, they are incomplete. Thus, a novel treatment option is required to suppress proliferation, invasion, angiogenesis, and migration of cholangiocarcinoma at the bile duct. We focused on the possibility of inhibiting proliferation, migration, invasion, and angiogenesis of cholangiocarcinoma using vorinostat and EGCG.

Vorinostat is an HDACi. Because histone acetylation facilitates loose chromatin structure and activates transcriptional potential, HDACs act as a transcription repressors and accelerate chromatin condensation [15]. HDACis such as vorinostat modify DNA transcription through chromatin remodeling and change the protein structure of transcription factor complexes. Vorinostat induces apoptosis, differentiation, proliferation inhibition, invasion inhibition and suppresses tumor cell angiogenesis potential [14, 15, 17, 30]. Takada et al. [14] reported that vorinostat potentiates apoptosis and inhibits invasion of various tumor cells by suppressing NF-*κ*B. As several genes including antiapoptotic, proliferative, and angiogenic products are regulated by NF-*κ*B, proliferation, invasion, and angiogenesis potential of tumor cells can be regulated or inhibited by vorinostat. Furthermore, vorinostat induces oxidative stress against various types of cancer cells [13, 15, 31]. Although cancer cells generate more ROS than those of normal cells, vorinostat induces further oxidative stress in tumor cells and enhances the suppression of cancer cell proliferation. In recent decades, the antitumor efficacy of vorinostat against tumor patients has been well demonstrated in clinical trials [32–36]. Kelly et al. reported that vorinostat with daily intravenous (i.v.) administration has antitumor efficacy against solid and hematological tumors and inhibits the biological target *in vivo* [32]. Furthermore, oral administration of vorinostat was also demonstrated to have antitumor activity in patients with advanced cancer [33, 34]. Clinical trials of vorinostat have been also performed against metastatic breast cancer and recurrent or metastatic head and neck cancer [35, 36]. Especially, vorinostat is also known to inhibit independently proliferation, invasion, and migration of glioma cells at 2D and 3D culture *in vitro* [37]. The potential of vorinostat against tumor patients with recurrent glioblastoma multiforme was also reported in clinical trials [38]. In our results, we also observed that vorinostat alone and vorinostat/EGCG combination induce inhibition of MMP-2/9 and invasionof HuCC-T1 cells (Figure 3 and Supplementary Figure 4 available online at doi: http://dx.doi.org/10.1155/2013/185158).

The principal merit of EGCG is to induce tumor cell apoptosis without adverse effect in normal cells. EGCG not only has a beneficial effect on tumor cell apoptosis but also inhibits tumor cell invasion and metastasis [18–27]. EGCG inhibits growth of prostate and breast tumors in nude mice [19, 20]. EGCG inhibits invasion and metastasis of tumor cells by regulating the activity of enzymes such as MMPs [25–27, 39]. EGCG inhibits migration, invasion, and spreading of melanoma cells dose dependently [39]. Furthermore, EGCG inhibits tyrosine phosphorylation of focal adhesion kinase and MMP-9 activity [40, 41]. As shown in Figure 3, our study showed that EGCG inhibited invasion and migration of HuCC-T1 human cholangiocarcinoma cells and suppressed MMP-9 activity.

This is the first report on the synergistic effect of vorinostat and EGCG for inhibiting proliferation, invasion, migration, and angiogenesis of human cholangiocarcinoma cells. In fact, vorinostat and EGCG have been frequently used in combination rather than as single agents [14, 31, 40–42]. Vorinostat has synergistic effects against various cancer cells in combinations with decitabine, tumor necrosis factor, cisplatin, 5-fluorouracil, doxorubicin, and paclitaxel [14, 31]. Nihal et al. [41] reported that the vorinostat and EGCG combination synergistically inhibits growth of melanoma cells by enhancing apoptosis and activating p21, p27, and caspases. The vorinostat and EGCG combination also downregulated the antiapoptotic protein Bcl-2 and upregulated proapoptotic protein Bax (Figure 2(c)). Lang et al. reported that EGCG accelerates apoptosis in human cholangiocarcinoma cells when used in combination with gemcitabine. As shown in Figure 2, we also found that the vorinostat and EGCG combination enhanced apoptotic signals, increased the Bax/Bcl-2 ratio, and promoted cleaved PARP expression level. Furthermore, the synergistic effects of drug combination were maintained for 72 hours (Supplementary Figure 1). The synergistic effects of induced apoptosis (Figure 2) and modulation of the HuCC-T1 cholangiocarcinoma cell cycle were observed (Supplementary Figure 2). p53 expression also increased following the combined vorinostat and EGCG treatment (Supplementary Figure 3).

 Vorinostat and EGCG have contrasting tumor cell oxidative stress behavior, as vorinostat induces ROS whereas EGCG is an antioxidant [31, 41]. EGCG is reported to complement vorinostat, as vorinostat activity decreased under high ROS levels in cell culture but markedly inhibits tumor cell growth at a low ROS level [43]. However, pretreatment with antioxidants reduces cellular ROS level and synergistically sensitizes tumor cell oxidative stress by vorinostat following higher tumor cell growth inhibition compared to that of a single vorinostat treatment. In our study, vorinostat alone induced increased cellular ROS but ROS level decreased significantly both in the EGCG only and vorinostat and EGCG combined treatments (Supplementary Figure 5). EGCG treatment to HuCC-T1 cells was resulted in a decrease in cellular ROS level compared to nontreated cells. The tendency for the ROS decrease was not reversibly changed by combined treatment of vorinostat and EGCG (Supplementary Figure 5), indicating that antioxidants effect of EGCG might be dominant in the molecular mechanism of oxidative stress in the cells. Basu et al. reported that pretreatment with antioxidants sensitizes growth inhibitory effect of vorinostat against oxidatively stressed human cancer cells [43]. They argued that vorinostat treatment markedly inhibits growth of LNCaP cells when the cells are at a low ROS and, however, potency of vorinostat was significantly decreased against the same cell line at high ROS level. Pretreatment of vitamin E as an antioxidant reduced cellular ROS level and then synergistically sensitized oxidatively stressed LNCaP cells [43]. They also demonstrated that the potency of vorinostat against cancer cells could be improved by combination with antioxidants. Furthermore, EGCG is known to stimulate cancer-specific induction of ROS and to induce preferential death of cancer cells [24]. Nihal et al., also reported that combination of EGCG and vorinostat results in significant inhibition of cell proliferation and increase in apoptotic signals [41]. Li et al. observed that estrogen receptor-*α* (ER*α*) expression in ER*α*-negative MDA-MB-231 breast cancer cells was synergistically reactivated by combination of EGCG and trichostatin A (TSA) [44]. They also found that EGCG/TSA combination was effective to inhibit HDACs activity and cell viability. Furthermore, they observed that EGCG alone significantly inhibited binding of HDAC1. Many investigations also reported that EGCG affects the activity of HDAC and histone acetyltransferase (HAT) [28, 41, 44–46]. Choi et al. identified EGCG as a potent HAT inhibitor (HATi) [45]. They observed that EGCG has global specificity for the majority of HAT enzymes but with negligible effect on the HDAC activity. Other reports showed that EGCG induced Raf kinase inhibitor protein (RKIP) upregulation via the inhibition of histone deacetylase (HDAC) activity [46].

Angiogenesis potential of HUVECs and VEGF expression in HuCC-T1 cholangiocarcinoma cells also decreased with the combined treatment of vorinostat and EGCG (Figure 4). HDACis such as vorinostat inhibit VEGF-induced expression of VEGF receptors in endothelial cells and then inhibit angiogenesis [15].

Our results demonstrated the synergistic effect of a combination of vorinostat and EGCG on suppressing proliferation, invasion, migration, angiogenesis, and induction of apoptosis. We suggest that vorinostat and EGCG together are a novel treatment option for CCA chemotherapy.

## Supplementary Material

Supplementary Figure 1 showed that the synergistic effect of vorinostat/EGCG combination on the proliferation and viability of HuCC–T1 cells. Combination of vorinostat and EGCG showed synergistic growth inhibition and cytotoxic effect against HuCC–T1 cells. Furthermore, less than 10 % of cells were remained 72 h after treatment of vorinostat/EGCG combination.
Supplementary Figure 2 showed the cell cycle arrest analysis of HuCC–T1 cells after treatment of vorinostat/EGCG combination. As shown in supplementary Figure 2, G1 phase was decreased and sub–G1 phase increased after treatment of vorinostat/EGCG combination.
Supplementary Figure 3 showed the p53 expression of HuCC–T1 cells after treatment of vorinostat/EGCG combination. Compared to single treatment of vorinostat or EGCG, combination of vorinostat/EGCG clearly enhanced p53 nuclear translocation in Hucc–T1 cells, indicating that combination of vorinostat/EGCG induced apoptosis of HuCC–T1 cells.
Supplementary Figure 4 showed the MMP2 and 9 expression of HuCC–T1 cells after treatment of vorinostat or EGCG. Both vorinostat and EGCG dose-dependently suppressed MMP–2 expression of HuCC–T1 cells even though vorinostat did not significantly affected MMP–9 expression of HuCC–T1 cells.
Supplementary Figure 5 showed the ROS generation after treatment of vorinostat and EGCG. As shown in supplementary Figure 5, vorinostat dose–dependently increased ROS generation of HuCC–T1 cells. However, EGCG significantly decreased ROS generation of HuCC–T1 cells until 200 min and then the level of ROS was slightly changed.
Interestingly, the ROS production of HuCC–T1 cells at treatment of vorinostat/EGCG combination was quite similar to the results of EGCG, indicating that EGCG has dominant effect on the ROS generation in the HuCC–T1 cells compared to vorinostat. These results indicated that vorinostat and EGCG combination showed synergistic effect on the proliferation, viability, cell cycle, apoptosis, MMP expression, and ROS generation of HuCC–T1 cells.

## Abbreviations

PARP: Poly (ADP-ribose) polymerase
MMP: Matrix metalloproteinase
EGCG: (−)-Epigallocatechin-3-gallate
HUVECs: Human umbilical vein endothelial cells
HDACi: Histone deacetylase inhibitor
VEGF: Vascular endothelial growth factor
NF-*κ*B: Nuclear factor-kappa B
TUNEL: Terminal deoxynucleotidyl transferase dUTP nick end labeling
ROS: Reactive oxygen species.
