# Supplementary material for: Synergistic Anticancer Effects of Vorinostat and Epigallocatechin-3-Gallate against HuCC-T1 Human Cholangiocarcinoma Cells
Source: Evid Based Complement Alternat Med. 2013 Jun 23;2013:185158. doi: 10.1155/2013/185158 (PMC3706064; doi:10.1155/2013/185158)
Supplement: Supplementary file 1 — Supplementary Figure 1 showed that the synergistic effect of vorinostat/EGCG combination on the proliferation and viability of HuCC–T1 cells. Combination of vorinostat and EGCG showed synergistic growth inhibition and cytotoxic effect against HuCC–T1 cells. Furthermore, less than 10 % of cells were remained 72 h after treatment of vorinostat/EGCG combination. Supplementary Figure 2 showed the cell cycle arrest analysis of HuCC–T1 cells after treatment of vorinostat/EGCG combination. As shown in supplementary Figure 2, G1 phase was decreased and sub–G1 phase increased after treatment of vorinostat/EGCG combination. Supplementary Figure 3 showed the p53 expression of HuCC–T1 cells after treatment of vorinostat/EGCG combination. Compared to single treatment of vorinostat or EGCG, combination of vorinostat/EGCG clearly enhanced p53 nuclear translocation in Hucc–T1 cells, indicating that combination of vorinostat/EGCG induced apoptosis of HuCC–T1 cells. Supplementary Figure 4 showed the MMP2 and 9 expression of HuCC–T1 cells after treatment of vorinostat or EGCG. Both vorinostat and EGCG dose-dependently suppressed MMP–2 expression of HuCC–T1 cells even though vorinostat did not significantly affected MMP–9 expression of HuCC–T1 cells. Supplementary Figure 5 showed the ROS generation after treatment of vorinostat and EGCG. As shown in supplementary Figure 5, vorinostat dose–dependently increased ROS generation of HuCC–T1 cells. However, EGCG significantly decreased ROS generation of HuCC–T1 cells until 200 min and then the level of ROS was slightly changed. Interestingly, the ROS production of HuCC–T1 cells at treatment of vorinostat/EGCG combination was quite similar to the results of EGCG, indicating that EGCG has dominant effect on the ROS generation in the HuCC–T1 cells compared to vorinostat. These results indicated that vorinostat and EGCG combination showed synergistic effect on the proliferation, viability, cell cycle, apoptosis, MMP expression, and ROS generatio [file 185158.f1.pdf]

Materials and Methods

Flow cytometry for cell cycle analysis

HuCC-T1 cells (1×10^6 cells/well) seeded in 6-well plates was exposed to various concentrations of vorinostat and EGCG combination for 24 hours. Then, the harvested cells were washed with PBS and fixed with 70% ethanol at -20°C for 1 hour. The fixed cells were centrifuged and washed with PBS three times. One unit of RNase A was added to cell suspension and incubated for 30min at 37°C. Fifty micro-liters of PI were added directly to the cell suspension, and the cells were analyzed by flow cytometry after 15 minutes.

p53 expression of tumor cells

Cells were grown on slides (Paul Marienfeld GmbH & Co., Lauda-Königshofen, Germany) in 6-well plates and then treated with EGCG, vorinostat, or their combination. After 24 hours, cells were fixed with 4% paraformaldehyde and permeabilized with 0.1% Triton-X 100 in PBS and then blocked for 1 hour at room temperature in 10% BSA in PBS. Then, anti-p53 (Santa Cruz Biotechnology, Santa Cruz, CA, USA) (1:250 in blocking solution) was added and incubated for 16 hours at 4°C. Alexa Fluor 488 (Invitrogen) (1:500 in blocking solution) was added and incubated for 1 hour at room temperature. The slides were washed with PBS and counterstained with 4,6-diamidino-2-phenylindole (DAPI; Santa Cruz Biotechnology). Cells were mounted and examined under a laser scanning confocal microscope (Olympus, Tokyo, Japan).

ROS measurement

ROS was measured as reported previously (Kim *et al*. 2011). After treatment, ROS generation in HuCC-T1 cells was measured by the DCFH-DA method. The fluorogenic substrate DCFH-DA, a cell permeable dye, is oxidized to highly fluorescent DCF by ROS, and can be used to monitor intracellular ROS generation. The cells were treated with vorinostat and EGCG for 24 hours in phenol red-free RPMI media. DCFH-DA was added to the media at a final concentration of 20 μM and incubated at 37°C for 30 minutes. After treatment at 635 nm, ROS generation was measured by fluorescence intensity at an excitation wavelength of 485 nm and an emission wavelength of 535 nm using a microplate reader.

Results

Supplementary Figure 1 showed that the synergistic effect of vorinostat/EGCG combination on the proliferation and viability of HuCC-T1 cells. Combination of vorinostat and EGCG showed synergistic growth inhibition and cytotoxic effect against HuCC-T1 cells. Furthermore, less than 10 % of cells were remained 72 h after treatment of vorinostat/EGCG combination.

Supplementary Figure 2 showed the cell cycle arrest analysis of HuCC-T1 cells after treatment of vorinostat/EGCG combination. As shown in supplementary Figure 2, G1 phase was decreased and sub-G1 phase increased after treatment of vorinostat/EGCG combination.

Supplementary Figure 3 showed the p53 expression of HuCC-T1 cells after treatment of vorinostat/EGCG combination. Compared to single treatment of vorinostat or EGCG, combination of vorinostat/EGCG clearly enhanced p53 nuclear translocation in Hucc-T1 cells, indicating that combination of vorinostat/EGCG induced apoptosis of HuCC-T1 cells.

Supplementary Figure 4 showed the MMP2 and 9 expression of HuCC-T1 cells after treatment of vorinostat or EGCG. Both vorinostat and EGCG dose-dependently suppressed MMP-2 expression of HuCC-T1 cells even though vorinostat did not significantly affected MMP-9 expression of HuCC-T1 cells.

Supplementary Figure 5 showed the ROS generation after treatment of vorinostat and EGCG. As shown in supplementary Figure 5, vorinostat dose-dependently increased ROS generation of HuCC-T1 cells. However, EGCG significantly decreased ROS generation of HuCC-T1 cells until 200 min and then the level of ROS was slightly changed. Interestingly, the ROS production of HuCC-T1 cells at treatment of vorinostat/EGCG combination was quite similar to the results of EGCG, indicating that EGCG has dominant effect on the ROS generation in the HuCC-T1 cells compared to vorinostat.

These results indicated that vorinostat and EGCG combination showed synergistic effect on the proliferation, viability, cell cycle, apoptosis, MMP expression, and ROS generation of HuCC-T1 cells.

Supplementary Figure legend

Supplementary Figure 1. Synergistic anticancer effect of vorinostat and epigallocatechin gallate (EGCG) over 48 or 72 hours. A 3×10^4 aliquot of cells for cell cytotoxicity assay and 3×10^3 cells for growth inhibition were seeded in 96-well plates. RPMI1640 media supplemented with 10% FBS were used for the tumor cell growth inhibition assay and serum-free media were used for cell cytotoxicity. * $p < 0.05$; ** $p < 0.01$; *** $p < 0.001$.

Supplementary Figure 2. The synergistic effect of a combination of vorinostat and epigallocatechin gallate (EGCG) was measured on HuCC-T1 cholangiocarcinoma cell cycle arrest. The combination increased apoptosis in comparison with vorinostat or EGCG alone. Propium iodide was used for cell cycle analysis. The sub-G1 phase was observed by treating the cells with the vorinostat and EGCG combination.

Supplementary Figure 3. Synergistic effect of the combination of vorinostat and epigallocatechin gallate (EGCG) enhanced p53 nuclear translocation in Hucc-T1 cells. HuCC-T1 cells were treated with vorinostat and EGCG for 24 hours. p53 nuclear translocation increased following the combined treatment. Images were captured using a 1200× objective on a laser scanning confocal microscope (Olympus, Tokyo, Japan).

Supplementary Figure 4. The effect of vorinostat and epigallocatechin gallate (EGCG) on migration capacity of HuCC-T1 cholangiocarcinoma cells. Matrix metalloproteinase 2 and 9 expression was measured by gelatin zymography. EGCG treatment alone significantly suppressed MMP-2 and 9 activity, whereas vorinostat alone mainly inhibited MMP-2.

Supplementary Figure 5. A single treatment of vorinostat alone induced increased cellular reactive oxygen species (ROS) level, which decreased significantly following the EGCG alone and the vorinostat/EGCG combined treatments. EGCG potentiated the decrease in cellular ROS level and the tendency for the ROS decrease did not significantly change with the vorinostat/EGCG combined treatment.

Reference

Kim, C.H., Chung, C.W., Choi, K.H., Yoo, J.J., Jeong, Y.I., Kang, D.H. 2011. Effect of 5-aminolevulinic acid-based photodynamic therapy via reactive oxygen species in himan cholangiocarcinoma cells. Int. J. Nanomed. 6, 1357-1363.

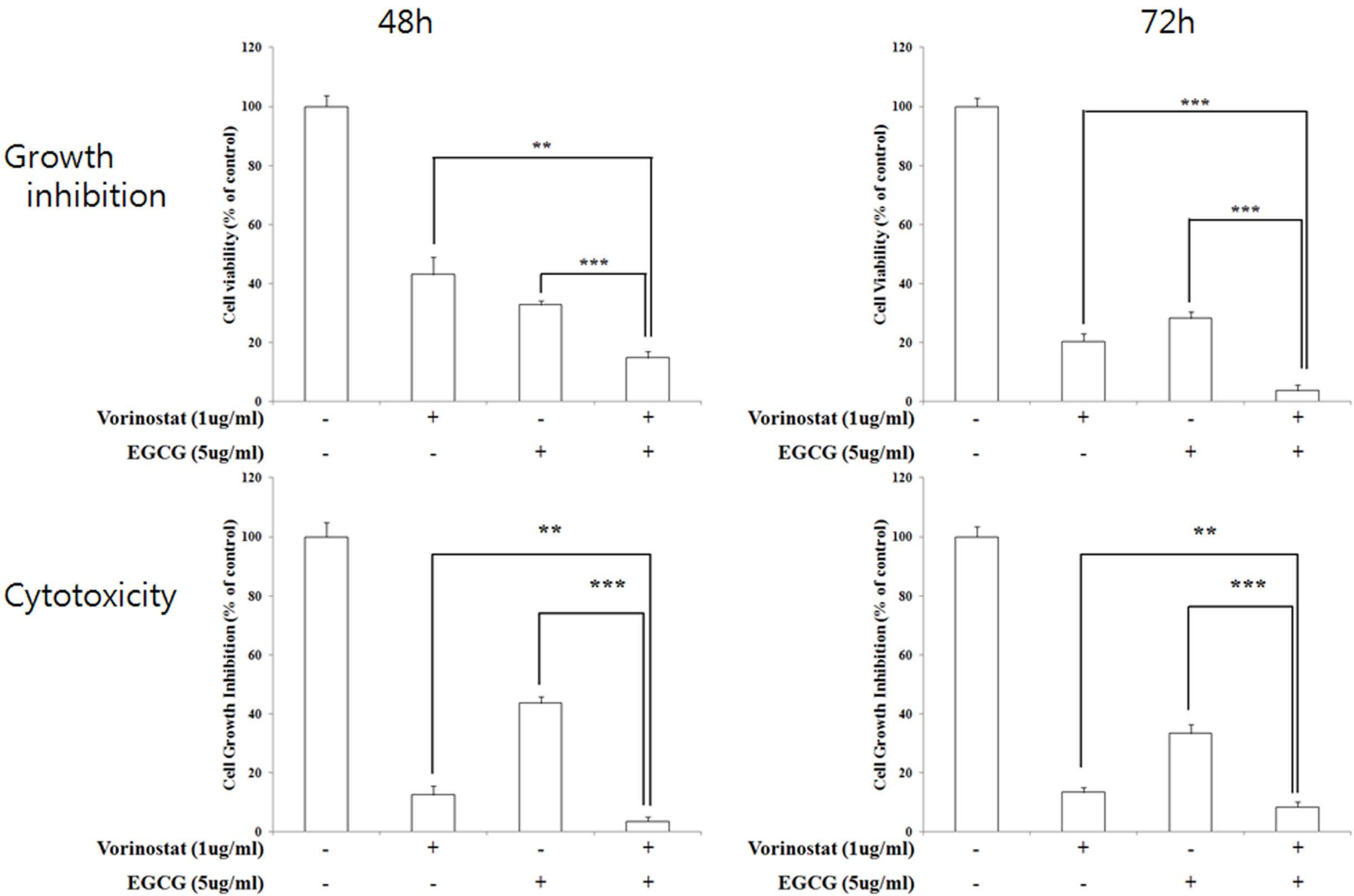

48h
72h
Growth inhibition
Cytotoxicity
Cell viability (% of control)
Cell Viability (% of control)
Cell Growth Inhibition (% of control)
0
20
40
60
80
100
120
**
***
Vorinostat (1ug/ml) - + - +
EGCG (5ug/ml) - - + +

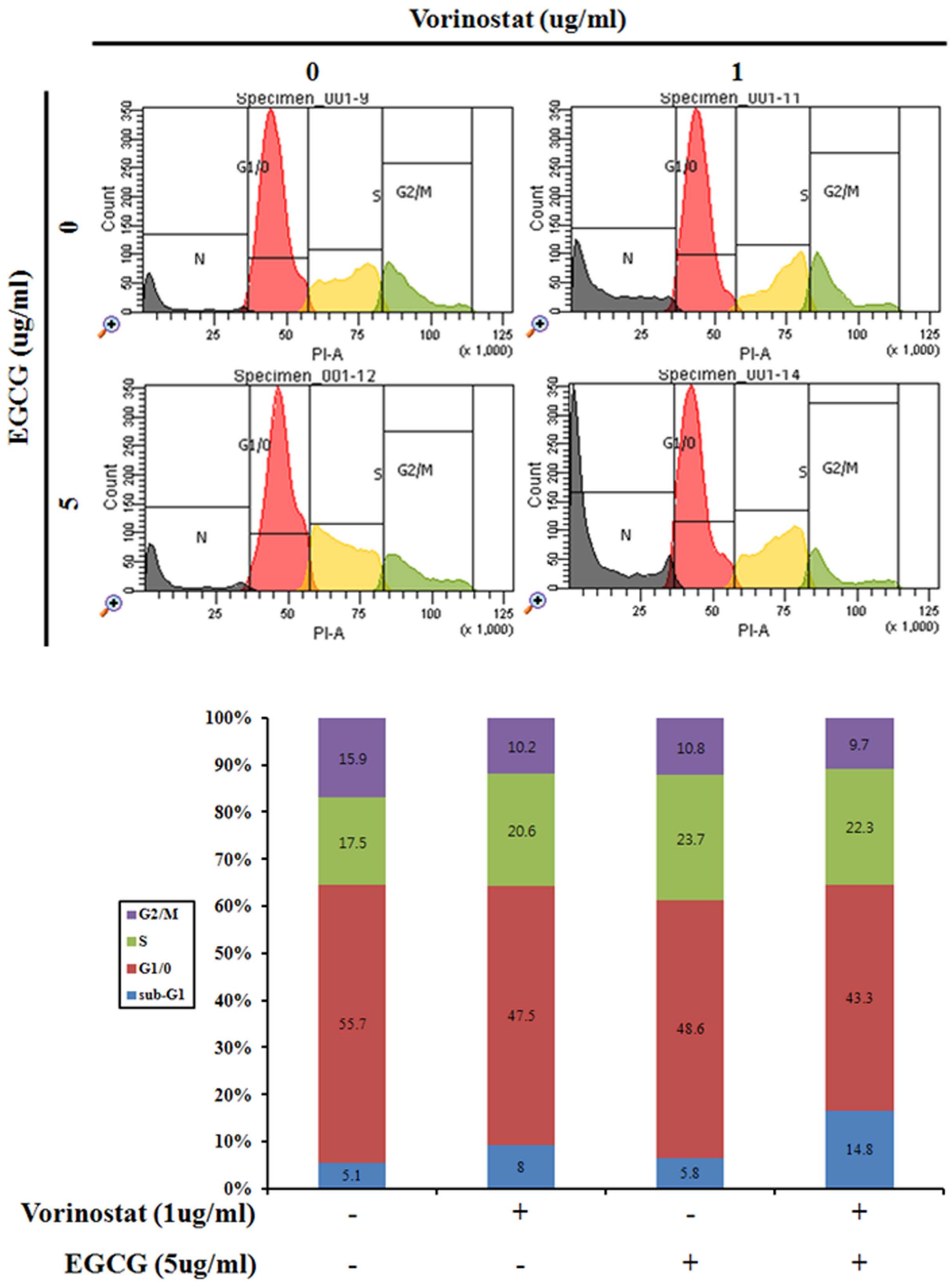

Vorinostat (ug/ml)
0
1
EGCG (ug/ml)
0
5
Specimen_001-9
Specimen_001-11
Specimen_001-12
Specimen_001-14
Count
PI-A
(x 1,000)
N
G1/0
S
G2/M
G2/M
S
G1/0
sub-G1
15.9
17.5
55.7
5.1
10.2
20.6
47.5
8
10.8
23.7
48.6
5.8
9.7
22.3
43.3
14.8
Vorinostat (1ug/ml)
-
+
-
+
EGCG (5ug/ml)
-
-
+
+

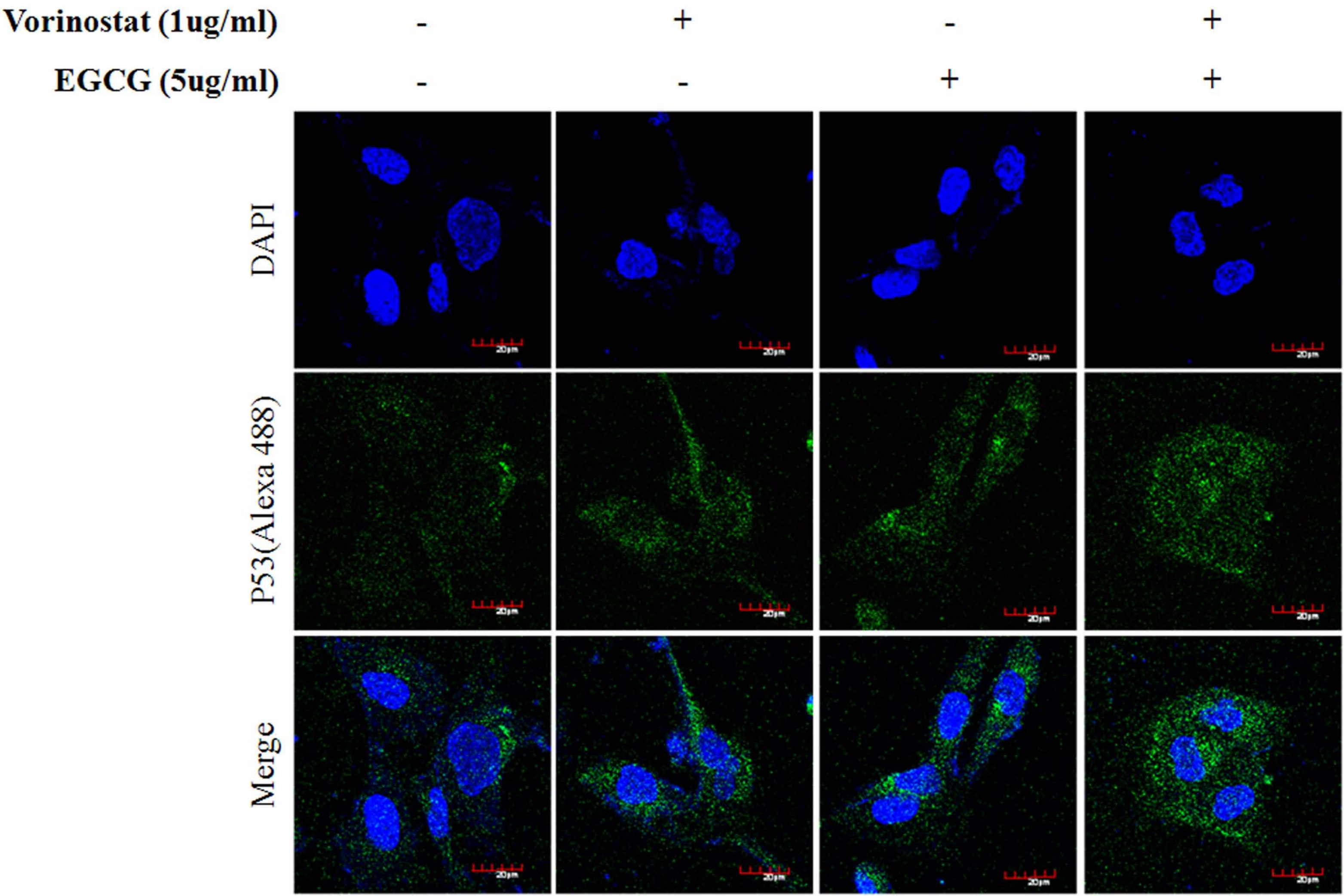

Vorinostat (1ug/ml) - + - +
EGCG (5ug/ml) - - + +
DAPI
P53(Alexa 488)
Merge

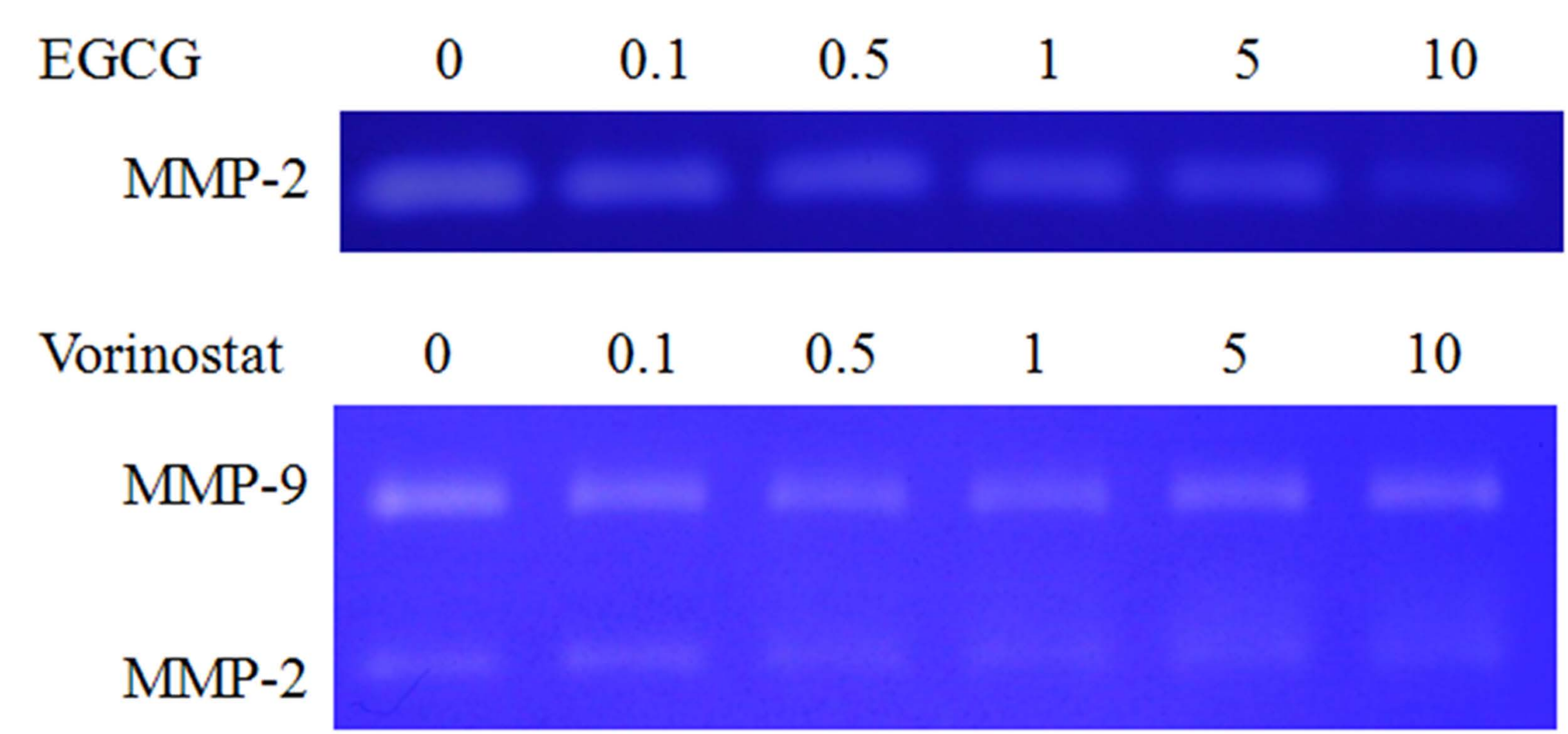
EGCG
0
0.1
0.5
1
5
10
MMP-2
Vorinostat
0
0.1
0.5
1
5
10
MMP-9
MMP-2

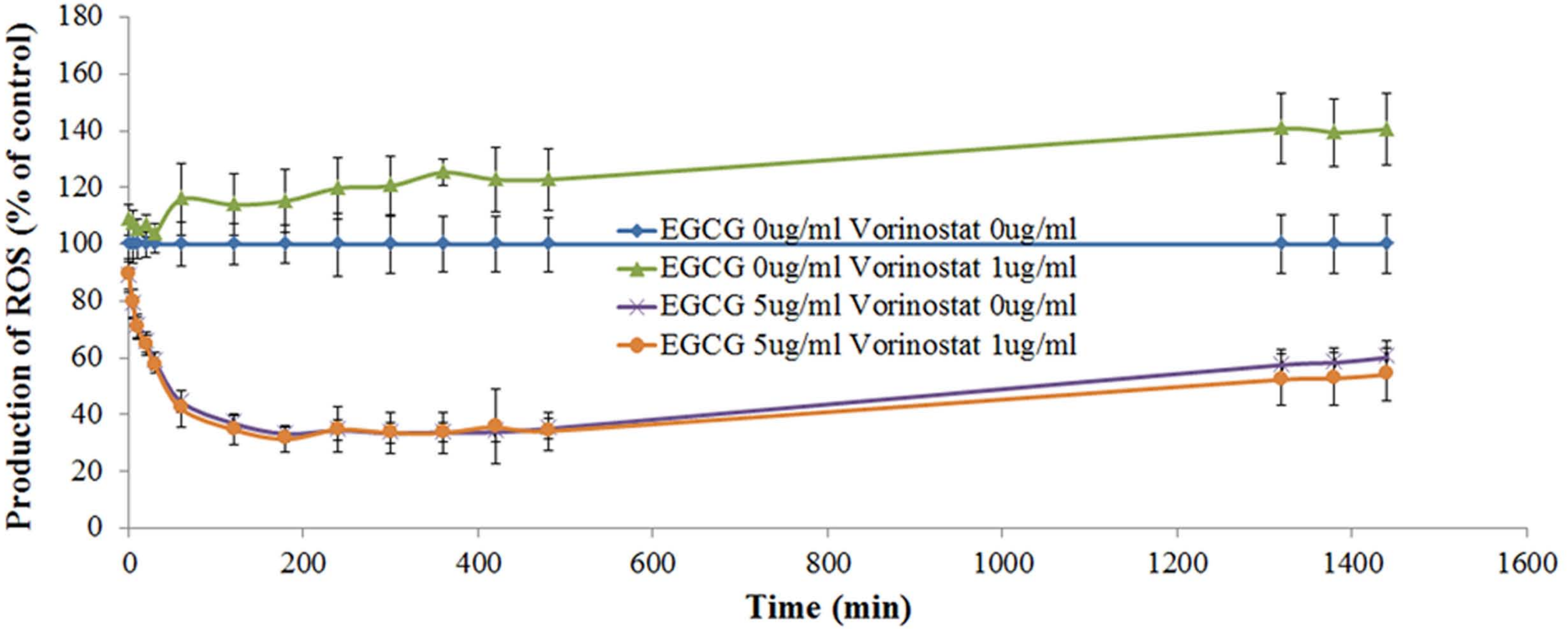

Production of ROS (% of control)
Time (min)
EGCG 0ug/ml Vorinostat 0ug/ml
EGCG 0ug/ml Vorinostat 1ug/ml
EGCG 5ug/ml Vorinostat 0ug/ml
EGCG 5ug/ml Vorinostat 1ug/ml
0
20
40
60
80
100
120
140
160
180
0
200
400
600
800
1000
1200
1400
1600

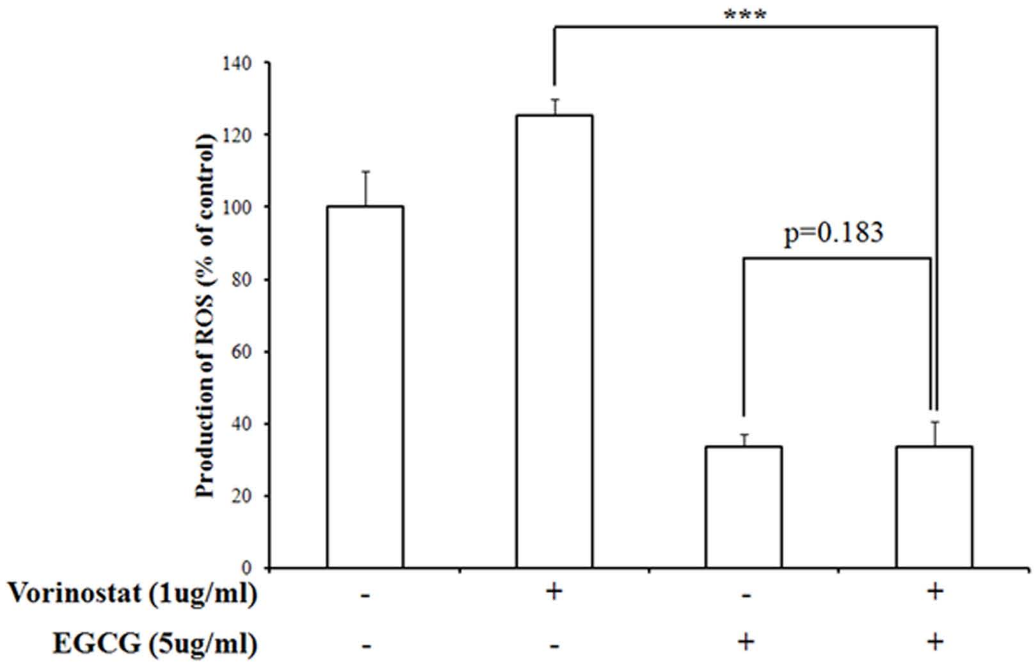

Production of ROS (% of control)
***
p=0.183
0
20
40
60
80
100
120
140
Vorinostat (1ug/ml) - + - +
EGCG (5ug/ml) - - + +
